# Cardiometabolic Biomarkers in Young Black Girls: Relations to Body Fatness and Aerobic Fitness, and Effects of a Randomized Physical Activity Trial

**DOI:** 10.1155/2011/219268

**Published:** 2011-10-11

**Authors:** Bernard Gutin, Ryan A. Harris, Cheryl A. Howe, Maribeth H. Johnson, Haidong Zhu, Yanbin Dong

**Affiliations:** ^1^Georgia Prevention Institute, Department of Pediatrics, Georgia Health Sciences University (Formerly the Medical College of Georgia), Augusta, GA 30912, USA; ^2^Department of Nutrition, University of North Carolina-Chapel Hill, Chapel Hill, NC 27514, USA; ^3^School of Applied Health Sciences and Wellness, Ohio University, Athens, OH 45701, USA; ^4^Department of Biostatistics, Georgia Health Sciences University, Augusta, GA 30912, USA

## Abstract

There is little evidence from randomized trials showing that physical activity alone influences biomarker profiles in youths. This study tested two hypotheses: (i) that elevated body fatness and poor fitness would be associated with unfavorable levels of cardiometabolic biomarkers in 8–12-y-old black girls (*n* = 242) and (ii) that a 10-mo PA intervention would have favorable effects on the fatness-related cardiometabolic biomarkers. At baseline, all fatness indices (i.e., percent body fat, visceral adipose tissue, BMI, and waist circumference) were significantly (*P* < 0.05) associated with unfavorable levels of insulin, glucose, systolic BP, diastolic BP, triglycerides, C-reactive protein (CRP), and fibrinogen. Aerobic fitness was significantly (*P* < 0.05) associated with favorable levels of insulin, CRP, fibrinogen, and HDL_2_. The PA intervention had significant and favorable effects on fitness, fatness, and two biomarkers—resting heart rate and LDL cholesterol. More research is needed to clarify what types of interventions can enhance the cardiometabolic health of youths.

## 1. Introduction

The relatively high prevalence of obesity and overweight in black females begins early in life [1]. Already in childhood, higher levels of general and visceral adiposity are associated with poor cardiometabolic biomarker profiles [2]. Moreover, black females are also at greater risk of exhibiting unfavorable levels of obesity-related cardiometabolic biomarkers that lead to hypertension [3], type 2 diabetes [4], and coronary heart disease [5]. Therefore, it is important to enhance our understanding of these relations and to develop effective prevention strategies to reduce future risk of these diseases, starting in childhood. 

A recent review of the literature on the effect of physical activity (PA) on cardiometabolic biomarker profiles [6] found evidence that PA, even without dietary intervention, leads to favorable changes in biomarker profile in obese youths who exhibit unfavorable levels prior to the intervention; however, there is little evidence from randomized trials showing that PA alone influences biomarker profiles in youths of all fatness levels, perhaps because the doses of PA used in previous randomized trials of nonobese youths have been rather modest. 

This study tested two hypotheses: (i) that lower levels of aerobic fitness and higher levels of fatness would be associated with poorer cardiometabolic biomarker profiles in a sample of young black girls who varied over the spectrum of fatness and fitness and (ii) that girls randomized to an after-school PA program that utilized a relatively large PA dose (i.e., 80 min/day of moderate-vigorous PA, 5 days/wk, 10 mo in duration) would exhibit beneficial changes in biomarkers compared to girls not randomized to the PA program. In addition, we explored two other questions dealing with individual differences over the 10-mo period of the intervention: (i) whether larger decreases in fatness and improvements in fitness would be associated with greater beneficial changes in biomarkers and (ii) whether, within the PA group, greater attendance and higher PA intensity, as measured with heart rate (HR) monitors during the PA sessions, would be associated with larger beneficial changes in the biomarkers.

## 2. Materials and Methods

### 2.1. Subjects

8–12-y-old black girls were recruited from local elementary schools using flyers. All black girls in third, fourth, and fifth grades were eligible if they met the following criteria: (i) weighed less than 300 lbs, the mechanical limit of the dual-energy-X-ray (DXA) machine (ii) were not taking any medications known to affect metabolism, body composition or fat distribution and (iii) were able to participate in regular PA. Subjects and their parents attended information sessions and signed informed consent/assent forms in accordance with the Human Assurance Committee of the Georgia Health Sciences University (GHSU). We found in previous studies that accepting only one sibling per family resulted in eligible and interested potential subjects refusing participation. Therefore, we decided to accept sisters to increase study acceptability on the part of subjects and their parents.

### 2.2. Testing

Subjects came to the Georgia Prevention Institute (GPI) for testing at the beginning of the study and after 10 mo. Subjects presented early in the morning after a 10-h fast. Pretesting started in late July and ended in mid-fall. Subjects were tested and if randomized to the intervention were integrated on a rolling basis. Subjects were paid $100 each for the baseline and posttest assessment.

### 2.3. Sexual Maturation

Pubertal stages were assessed by pediatricians based on established criteria [7]. Examination of the Tanner staging for breast and pubic hair development was not refused by any of the subjects. None of the children had hirsutism.

### 2.4. Body Size and Composition

Height (cm) and weight (kg) were measured by standard methods using a wall-mounted stadiometer and a scale, respectively. Body mass index (BMI) was calculated as weight (kg)/height (m^2^), and BMI percentile was obtained from growth charts from the Centers for Disease Control [8]. Waist circumference (cm) was measured using a measuring tape at the narrowest point of the torso, below the rib cage and above the umbilicus. Total body composition, including %BF, was obtained using DXA (Hologic QDR-1000, Waltham, Mass, USA) as previously described [9]. Visceral adipose tissue (VAT) was obtained using magnetic resonance imaging (MRI: 1.5 T General Electric Medical Systems, Milwaukee, Wis, USA), as previously described [10]. The MRI equipment in the hospital was not available at all times when subjects were being tested; therefore, VAT was only available on a subset of subjects.

### 2.5. Hemodynamics

Resting hemodynamic measures were made in the supine position after 10 min of quiet rest with arms at the side of the body and legs uncrossed. Systolic and diastolic BP (SBP and DBP, resp.) and heart rate (HR) were measured with a Dinamap monitor (Critikon, Inc., Tampa, Fla, USA); five readings were made at 1-min intervals and the last three were averaged.

### 2.6. Bioassays

Fasting blood samples were obtained from participants. Serum was sent to the University of Alabama (UAB) for the determination of fasting insulin and glucose levels. Glucose was measured in 10 uL sera using an Ektachem DT II System (Johnson and Johnson Clinical Diagnostics, Rochester, NY, USA). The intraassay coefficient of variation was 0.61%; mean interassay coefficient of variation was 1.45%. Insulin was assayed in duplicate 100 uL aliquots of sera by specific radioimmunoassay (Linco Research Inc., St. Charles, MO); cross-reactivity with proinsulin is less than 0.2%. Assay sensitivity was 3.41 uU/mL; intra-assay coefficient of variation was 3.7%.

Plasma concentrations of triglycerides (TG), total cholesterol (TC), low-density lipoprotein cholesterol (LDLC), and high-density lipoprotein cholesterol (HDLC) were measured at the Emory Lipid Research Laboratory using homogeneous enzymatic assays (Equal Diagnostics, Exton, Pa, USA). Plasma concentrations of Apolipoprotein(Apo)A1, ApoB, and lipoprotein(a) (Lp(a)) were determined using immunoturbidimetric methods (DiaSorin, Stillwater, Minn, USA). ApoCIII was determined using an immunoturbidometric assay kit (Wako Chemicals, Richmond, Va, USA). ApoE was determined on the Beckman CX7 Chemistry analyzer by immunoturbidometric method using the kit from Wako Chemicals (Richmond, Va, USA). The segmental gradient polyacrylamide gels (S-GGE 2.8/8.30) were prepared for the determinations of LDL and HDL_2_ diameters [11]. Inflammatory and coagulation markers were assayed in the Inflammatory Core Lab of the GHSU. Serum C-reactive protein (CRP) was assayed in duplicate using ELISAs. Fibrinogen was measured using citrated plasma and was assayed in duplicate using a BBL Fibrometer and reagents purchased from Biomerieux, (St. Louis, Mo, USA) as previously described [2].

### 2.7. Aerobic Fitness

 Fitness was assessed using a multistage treadmill test. Heart rate was monitored using a Polar Accurex Plus or S610 HR monitor (Lake Success, Long Island, NY, USA). Oxygen consumption (VO_2_) was measured using a Sensormedics Vmax 229 cardiopulmonary system (Yorba Linda, Calif, USA). The treadmill protocol began with a 4-min warm-up at 0% grade and 2.0 mph. The speed was then increased 0.5 mph every 2 min until reaching 3.0 mph at which time the grade increased to 2% for 2 min, then increased an additional 3% every 2 min until reaching 20% grade or exhaustion. Two indices of aerobic fitness were obtained: maximal oxygen consumption (VO_2_ max) and oxygen consumption at a HR of 170 bpm (VO_2_-170). Subjects were considered to have attained VO_2_ max if they met two of the following three criteria: (i) an increase in HR <5 bpm between the final two workloads (ii) an increase in VO_2_ <100 mL between the final two workloads and (iii) a respiratory exchange ratio >1.00. Although all subjects were given verbal cues to give a maximal effort, about half of them stopped the test voluntarily before reaching VO_2_ max, both at baseline and after the 10-mo intervention period. In addition, only about half of the subjects who attained VO_2_ max at baseline also did so at postintervention. Using VO_2_ max as our index of fitness would have resulted in a 50% decrease in sample size for the analyses, greatly affecting power to detect significant changes and differences between groups. Therefore, a submaximal index of fitness (VO_2_-170) was used. Using all the treadmill workloads completed, we computed individual regression equations of VO_2_ on HR for each subject to obtain VO_2_-170.

### 2.8. After-School Intervention

The after-school intervention is described in detail elsewhere [12]. Briefly, after pre-testing, subjects within each school were randomized 3 : 2 to the intervention or control groups, respectively. We randomized a larger number to the intervention group, because we planned to explore relations between biomarker changes and actual doses of PA achieved by the girls (i.e., attendance and HR during the PA sessions). The control group received no intervention. The intervention group stayed at their school at the end of the day every day that school was in session. In order to facilitate participation in the study, transportation home was provided to the subjects. The intervention consisted of 30 min of homework time during which subjects were provided with a healthy snack free of charge, and 80 min of PA. The PA component included 25 min of skills development, 35 min of vigorous PA, and 20 min of toning and stretching. Subjects wore Polar Accurex Plus or S610 HR monitors (Lake Success, Long Island, NY, USA) every day during the PA portion of the program and were taught how to maintain their HR above 150 bpm during the 35-min vigorous PA component of the intervention. Subjects received small weekly prizes (e.g., bouncy balls, slinkies, pencils, note pads, etc.) for maintaining good behavior, attitude, and attendance.

### 2.9. Statistical Analysis

All variables were checked for normality of distribution using normal probability plots prior to the analyses. The relationships of biomarkers with indices of fatness and fitness at baseline were analyzed using Spearman rank correlations to account for the nonnormal distributions of some of the variables. No transformations were needed for the changes from baseline. ANCOVA was performed to identify significant effects of the intervention on changes from baseline while adjusting for baseline values. Additional covariates analyzed were age and sexual maturation. Interactions between covariates and treatment group were explored. Only significant factors were retained in the model. The difference between the adjusted change scores reflects change in the intervention group relative to change in the control group. A negative difference is a relative decrease in comparison with controls even though both groups might have shown an increase from baseline to 10-mo due to growth. To investigate the effects of changes in fatness and fitness on changes in biomarkers, we used Pearson correlations. To investigate the effect of attendance to the program or intensity of training on changes in biomarkers, we included percent attendance or HR during the aerobic portion of the intervention in an analysis that also included baseline biomarker concentration, age, and sexual maturity. Statistical significance was set at alpha = 0.05. SAS version 9.2 was used for all analyses.

## 3. Results

The baseline characteristics for all the subjects are presented in Table 1. Although the subjects were not selected based on level of fatness, 21% were overweight and 31% were obese [12]. Nonetheless, baseline values of biomarkers were relatively normal. None of the subjects had fasting glucose values that would have labeled them with a provisional diagnosis of diabetes (≥7.0 mmol/L [13]). Seven percent had impaired fasting glucose (5.6–6.9 mmol/L), and 93% had normal values (<5.6 mmol/L). Values for BP were also relatively normal: 5% had SBP values above the 95th percentile for age, sex, and height [14, 15], 1% had DBP values above the 95th percentile for age, sex, and height, and <1% had both SBP and DBP values above the 95th percentile.

Table 2 shows the baseline correlations of fatness indices (%BF, VAT, BMI, and waist circumference) and aerobic fitness with each of the biomarkers. In the interests of parsimony, and because of the intercorrelations among some of the biomarkers, we omitted several from the correlation analyses. Higher levels of the fatness indices were significantly associated with less favorable levels of all of the biomarkers except diastolic BP; the correlations of BMI with LDLC and apoB1 did not reach the 0.05 level of significance (*P* < 0.10). Higher levels of aerobic fitness were associated with favorable levels of insulin, systolic BP, TG, CRP, and fibrinogen. In general, the fatness-biomarker correlations were substantially higher than the fitness-biomarker correlations.

Table 3 shows the baseline and postintervention biomarker values for the intervention and control groups, in which all intervention subjects were included regardless of their attendance at the after-school sessions. There were no significant differences between groups at baseline for any of the biomarkers. Also shown are the adjusted change values between the two groups. The baseline value of each dependent variable was associated with its change value. For example, those with a higher resting HR at baseline had a larger decrease in HR over the 10-mo period. Significant intervention effects were found only for resting HR and LDLC. There was a slight trend for a favorable intervention effect on TC and CRP (*P*'s < 0.10). Similar results were obtained when efficacy analyses were performed that included only subjects who came at least 2 days/wk (data not shown). 

Changes in body composition and fitness of the girls in this project were presented previously [12]. With respect to the fatness and fitness variables of particular interest to the current paper, effectiveness analyses showed that the intervention group (*n* = 118), compared to the control group (*n* = 83) had significant (*P* < 0.05) beneficial changes in %BF (−2.01), VAT (−14.6 cm^3^), BMI (−0.53), waist circumference (−1.68 cm), and aerobic fitness (1.57 mL/kg/min).

 The correlations between 10-mo changes in fatness and fitness, and changes in the biomarkers for all subjects from both groups are presented in Table 4. Greater improvements in fitness were significantly associated with greater increases in HDLC and lesser changes in fibrinogen. In addition, greater increases in %BF were significantly associated with greater increases in insulin and LDLC. Further, greater increases in BMI were significantly associated with greater increases in insulin, and fibrinogen. The highest correlation coefficients were shown for the relations between increases in VAT and increases in CRP and fibrinogen.

To obtain information about the dose-response relations between the PA and the changes in biomarkers, we examined the relations, within the intervention group, between changes in the biomarkers and two aspects of PA-dose-HR during the aerobic portion of the session as an index of intensity, and attendance at the PA sessions. Heart rate during the PA sessions was not significantly associated with changes in any of the biomarkers. However, there was a significant negative relationship between PA-attendance and change in fasting insulin (*β* = −0.70, *P* < 0.05). Subjects who attended the program less than 60% of the time exhibited increases in fasting insulin whereas those who attended at least 60% of the time had no change or a slight decrease in fasting insulin.

## 4. Discussion

Children in Georgia have been shown to be heavier than the national average [16]; therefore, it was not surprising to find that the sample in this study tended to be on the heavier side despite not having been recruited on the basis of fatness. Nonetheless, baseline values of the cardiometabolic biomarkers fell largely within normal ranges for girls in the 8–12 y range of age. Specifically, this was the case for fasting glucose [13] and BP [14, 15], as mentioned above, as well as for the lipids (TG, TC, HDLC, and LDLC) [17], CRP [18], and fibrinogen [18]. Although there are no reference values for the apolipoproteins or LDL size for children, the values in this sample of black girls are similar to those found in other studies of relatively healthy children [19, 20]. 

The descriptive baseline findings confirmed our first hypothesis: the fatness indices were associated with unfavorable values of almost all of the biomarkers investigated. Higher aerobic fitness was associated with more favorable levels of several of the biomarkers albeit with smaller correlations than the fatness correlations. This is consistent with the results of recent pediatric studies showing: (i) that fatness and fitness are correlated with unfavorable biomarker profiles in young children [21], and (ii) that cardiometabolic biomarkers are more closely associated with fatness than with fitness [6]. It is noteworthy that for both fatness and fitness the correlations were especially high for insulin and CRP, suggesting that insulin metabolism and inflammatory processes are especially sensitive to variations in lifestyle.

Although the intervention imparted a substantial dose of PA on the girls and had a significant impact on their fatness and fitness, our second hypothesis was not fully supported: we found few significant effects of the PA intervention on biomarker values. This result raises questions about the value of PA alone to improve the cardiometabolic biomarker profiles of black girls in the general population who are not preselected to have unfavorable baseline profiles. These findings also demonstrate the important distinction between observational studies and randomized trials. If, in fact, there is a cause-effect relationship between fatness and biomarker profiles, it may take longer than 10 mo for the PA-induced changes in fatness to be translated into biomarker changes. 

 It was noteworthy that favorable decreases in resting HR and LDLC were seen in the intervention group, especially given that the levels of both of these variables were within normal ranges for the majority of children at baseline. These results are consistent with the results of previous PA trials involving the general population of youths [6]. Additionally, changes in total cholesterol and CRP following the intervention were marginally significant (*P* < 0.10). Other studies of PA in children and adolescents, including overweight adolescents who had significant decreases in fatness, failed to show beneficial changes in CRP [22–24]; however, these interventions may have been too short—eight and twelve weeks—to show beneficial effects. Taken together, these results suggest that beneficial changes in some cardiometabolic biomarkers may occur with PA even within children whose levels are relatively normal for their age, provided that the PA is of sufficient intensity and duration. 

We failed to show that PA intensity was associated with change in biomarker levels within the intervention group. This may be because we tried to keep all the children working at a relatively high intensity, rather than allowing intensity to vary. Hence, there may not have been enough variability in intensity to reveal relationships. In a meta-analysis study of adults, LDLC was the only lipid or lipoprotein associated with training intensity [25, 26]. Furthermore, the only biomarker that we found to be associated with frequency of attendance was fasting insulin. 

One way to explain the discrepancy between the results of observational and intervention studies is to consider that the observational studies provide information about a relationship that has evolved over a long period of time; for example, youths who have been sedentary over several years before the measurements have gradually worsened their body composition and biomarker profile, with the result that a relationship between high levels of fatness and poor biomarker profile is clearly observed. Similarly, prospective observational studies that are carried out over several years time into the adult years may show that changes in PA are accompanied by worsening biomarker profile [15]. Because of the difficulty of conducting randomized controlled trials over such long periods of time, conclusive evidence from intervention trials is quite difficult to obtain. 

Our findings concerning the relations of changes in fatness to changes in biomarkers over the 10-mo duration of the trial are provocative and may suggest hypotheses to be further tested in future studies. Lesser increases in VAT were most highly correlated with favorable changes in CRP and fibrinogen. These results are consistent with the suggestion that visceral adiposity is an especially important influence on some aspects of cardiometabolic health [2, 27]. 

This study had several notable strengths. First, it focused on a specific demographic group—black females—who are at elevated risk of obesity and obesity-associated diseases; this focus enabled us to use physical activities in the PA sessions that we had previously found to be especially enjoyable to black girls. Second, the subjects were relatively young, enabling us to show substantial relations of fatness and fitness to biomarker profile early in life. Third, we measured general and visceral adiposity with high-technology imaging techniques, and aerobic fitness with a multistage treadmill test. Fourth, we investigated a wide variety of biomarkers, enabling us to explore which ones were more or less sensitive to variations in fatness and fitness, and sensitive to the influence of a PA intervention. Perhaps most important, we determined the results of a randomized trial that involved a relatively large dose of PA over a relatively long period of time. 

One limitation of the study concerns the selection of the biomarkers. Recent research has identified other inflammatory biomarkers that may be responsive to PA interventions, such as IL-6, TNF*α* as well as anti-inflammatory markers (i.e., IL-4 and IL-10) [28, 29]; these should be investigated in future PA studies of youths.

## 5. Conclusion

This study showed that lifestyle factors such as high body fatness and low aerobic fitness were associated with unfavorable levels of many cardiometabolic biomarkers in young black girls. However, a 10-mo intervention, that had favorable effects on body composition and fitness, had significant effects on only a small number of the biomarkers. These results highlight the important differences between observational designs and randomized controlled trials. More research is needed in which various doses of PA are imparted to youths over periods of several years to uncover the long-term relations of fatness and fitness to cardiometabolic health in youths.

## Figures and Tables

**Table 1:** Baseline subject characteristics.

	Control		Intervention		
	*N*	Mean (SD)	*N*	Mean (SD)	*P* value
Age (yrs)	102	9.6 (0.9)	140	9.5 (0.9)	0.31
Pubertal Status	97	2.3 (1.1)	134	2.1 (1.0)	0.11
Height (cm)	102	141.7 (9.4)	140	140.5 (9.4)	0.33
Weight (kg)	102	42.9 (15.1)	140	42.8 (13.8)	0.95
Waist (cm)	102	66.9 (11.7)	139	67.3 (11.6)	0.76
BMI (kg/m^2^)	102	21.0 (5.7)	140	21.3 (5.1)	0.64
Body Fat (%)	100	30.4 (12.6)	135	31.1 (11.7)	0.68
VO_2_-170 (mL/kg/min)	94	21.2 (4.9)	129	21.0 (5.2)	0.74

Means (±SD). SD: standard deviation; BMI: body mass index; VO_2_-170: oxygen consumption at 170 beats/min.

**Table 2:** Baseline Spearman correlations (*P* values) between aerobic fitness and fatness indices with cardiometabolic biomarkers.

	VO_2_-170 (mL/kg/min)	%BF	VAT (cm^3^)	BMI (kg/m^2^)	Waist (cm)
Insulin, pmol/L	−0.40 (<0.0001)	0.56 (<0.0001)	0.56 (<0.0001)	0.60 (<0.0001)	0.63 (<0.0001)
Glucose, mmol/L	−0.10 (0.18)	0.32 (<0.0001)	0.30 (0.0002)	0.25 (0.0004)	0.33 (<0.0001)
SBP, mm Hg	−0.15 (0.03)	0.26 (<0.0001)	0.19 (0.01)	0.27 (<0.0001)	0.25 (<0.0001)
DBP, mm Hg	−0.06 (0.39)	0.12 (0.06)	0.12 (0.09)	0.08 (0.19)	0.09 (0.18)
TG, mmol/L	−0.18 (0.02)	0.31 (<0.0001)	0.30 (0.0004)	0.33 (<0.0001)	0.39 (<0.0001)
LDLC, mmol/L	−0.07 (0.35)	0.19 (0.01)	0.22 (0.01)	0.14 (0.054)	0.16 (0.03)
ApoB, mg/dL	−0.13 (0.09)	0.19 (0.01)	0.21 (0.01)	0.12 (0.09)	0.15 (0.04)
ApoCIII, ng/mL	−0.04 (0.64)	0.29 (<0.0001)	0.26 (0.002)	0.26 (0.0003)	0.35 (<0.0001)
HDLC, mmol/L	0.12 (0.14)	−0.25 (0.0006)	−0.29 (0.0004)	−0.30 (<0.0001)	−0.34 (<0.0001)
CRP, ng/mL	−0.46 (<0.0001)	0.73 (<0.0001)	0.68 (<0.0001)	0.63 (<0.0001)	0.62 (<0.0001)
Fibrinogen, mg/dL	−0.28 (0.0002)	0.37 (<0.0001)	0.37 (<0.0001)	0.35 (<0.0001)	0.32 (<0.0001)

SBP: systolic blood pressure; DBP: diastolic blood pressure; TG: triglycerides; LDLC: low-density lipoprotein cholesterol; Apo: apolipoprotein; HDLC: high-density lipoprotein cholesterol; CRP: C-reactive protein.

**Table 3:** Cardiometabolic biomarkers at baseline and at 10 months for the intervention and control groups with all intervention subjects included regardless of attendance at the PA sessions (unadjusted mean [SD]).

Variable	Baseline		10 Months		Adjusted change (SE)*	*P* value
	Intervention	Control	Intervention	Control		
Insulin, pmol/L^†^	119.4 (84.8)	101.4 (59.9)	130.6 (79.7)	122.4 (57.1)	−3.6 (8.9)	0.69
Glucose, mmol/L^†^	4.89 (0.39)	4.91 (0.43)	4.85 (0.37)	4.89 (0.38)	−0.03 (0.06)	0.63
SBP, mm Hg^‡^	104.1 (7.5)	102.4 (9.1)	105.1 (8.3)	103.8 (7.6)	0.5 (1.0)	0.64
DBP, mm Hg^‡^	59.0 (5.6)	59.5 (5.7)	58.3 (6.5)	58.8 (5.4)	−0.3 (0.8)	0.72
HR, bpm^‡^	79.6 (9.2)	79.8 (8.9)	74.1 (8.9)	76.9 (8.3)	−2.7 (1.1)	0.01
TC, mmol/L^†^	4.18 (0.83)	4.15 (0.70)	4.01 (0.74)	4.15 (0.67)	−0.15 (0.09)	0.08
TG, mmol/L^†^	0.66 (0.30)	0.63 (0.30)	0.77 (0.37)	0.71 (0.32)	0.04 (0.05)	0.46
HDLC, mmol/L^†^	1.34 (0.32)	1.29 (0.28)	1.36 (0.31)	1.34 (0.31)	−0.01 (0.04)	0.73
LDLC, mmol/L^†^	2.67 (0.85)	2.67 (0.69)	2.47 (0.70)	2.67 (0.75)	−0.20 (0.10)	0.04
LP(a), mg/dL^†^	45.5 (30.1)	50.6 (42.8)	47.8 (31.1)	57.3 (48.1)	−4.48 (3.59)	0.21
LDL size, nm^†^	26.1 (0.69)	26.2 (0.69)	25.7 (0.82)	25.8 (0.72)	−0.09 (0.14)	0.50
%HDL_2_^†^	36.9 (20.3)	36.7 (20.2)	36.1 (20.3)	35.8 (17.6)	0.17 (3.30)	0.96
ApoA1, mg/dL^†^	132.8 (32.3)	124.3 (22.7)	126.2 (22.1)	125.3 (20.7)	−1.31 (3.68)	0.72
ApoB, mg/dL^†^	77.6 (29.9)	77.3 (26.1)	71.9 (26.6)	74.1 (21.9)	−2.3 (4.3)	0.60
ApoCIII, ng/mL^†^	12.0 (3.8)	11.4 (3.9)	11.1 (4.2)	11.5 (4.3)	−0.62 (0.69)	0.37
ApoE, ng/mL^†^	5.26 (1.49)	5.03 (1.75)	5.04 (1.56)	4.78 (1.56)	0.09 (0.20)	0.66
CRP, ng/mL^†^	757.3 (1571.0)	694.2 (883.6)	597.9 (1333.7)	795.8 (1120.5)	−245.8 (145.2)	0.09
Fibrinogen, mg/dL^†^	2.72 (0.51)	2.77 (0.55)	2.89 (0.50)	2.91 (0.60)	0.02 (0.08)	0.83

SD: standard deviation; SBP: systolic blood pressure; DBP: diastolic blood pressure; HR: heart rate; TC: total cholesterol; TG: triglycerides; HDLC: high-density lipoprotein cholesterol; LDLC: low-density lipoprotein cholesterol; Lp(a): lipoprotein(a); VLDL: very low-density lipoprotein; Apo: apolipoprotein; CRP: C-reactive protein.

*Change estimates and standard errors (SE) are the differences between intervention and control groups adjusted for age and baseline value.

^†^Intervention *N* = 74, Control *N* = 60.

^‡^Intervention *N* = 118, Control *N* = 82.

**Table 4:** Pearson correlations (*P* values) between changes in fatness and fitness indices and changes in cardiometabolic biomarkers.

	VO_2_-170 (mL/kg/min)	%BF	VAT (cm^3^)	BMI (kg/m^2^)	Waist (cm)
Insulin, pmol/L	−0.16 (0.1)	0.23 (0.01)	0.01 (0.92)	0.32 (0.0002)	0.19 (0.03)
Glucose, mmol/L	0.09 (0.35)	0.03 (0.72)	0.05 (0.66)	0.10 (0.24)	0.06 (0.48)
SBP, mm Hg	0.04 (0.60)	−0.02 (0.74)	−0.01 (0.95)	−0.017 (0.81)	0.05 (0.50)
DBP, mm Hg	−0.01 (0.89)	−0.07 (0.35)	−0.04 (0.67)	−0.11 (0.11)	−0.05 (0.52)
TG, mmol/L	−0.03 (0.79)	0.06 (0.48)	−0.17 (0.19)	−0.001 (0.99)	−0.06 (0.50)
LDLC, mmol/L	−0.18 (0.07)	0.22 (0.01)	0.20 (0.11)	0.14 (0.13)	0.08 (0.35)
ApoB, mg/dL	−0.12 (0.22)	0.07 (0.45)	−0.005 (0.97)	0.09 (0.33)	−0.01 (0.93)
ApoCIII, ng/mL	0.04 (0.66)	−0.01 (0.88)	0.10 (0.45)	−0.03 (0.73)	0.10 (0.27)
HDLC, mmol/L	0.21 (0.03)	−0.07 (0.46)	−0.09 (0.47)	−0.08 (0.35)	−0.05 (0.54)
CRP, ng/mL	−0.16 (0.13)	0.08 (0.43)	0.46 (0.0004)	0.04 (0.69)	0.12 (0.20)
Fibrinogen, mg/dL	−0.22 (0.02)	0.14 (0.12)	0.39 (0.001)	0.17 (0.05)	0.23 (0.008)

SBP: systolic blood pressure; DBP: diastolic blood pressure; TG: triglycerides; LDLC: low-density lipoprotein cholesterol; Apo: apolipoprotein; HDLC: high-density lipoprotein cholesterol; CRP: C-reactive protein.
